# An estimation of total antimicrobial usage in humans and animals in Vietnam

**DOI:** 10.1186/s13756-019-0671-7

**Published:** 2020-01-14

**Authors:** Juan J. Carrique-Mas, Marc Choisy, Nguyen Van Cuong, Guy Thwaites, Stephen Baker

**Affiliations:** 1 Oxford University Clinical Research Unit, Ho Chi Minh City, Vietnam; 2 Centre for Tropical Medicine and Global Health, Nuffield Department of Clinical Medicine, Oxford University Clinical Research Unit, 764 Vo Van Kiet, Ward 1, District 5, Ho Chi Minh City, Vietnam; 3 MIVEGEC, IRD, CNRS, University of Montpellier, Montpellier, France; 4 LMI “Drug Resistance in South-east Asia” (DRISA), Hanoi, Vietnam; 5 Cambridge Institute of Therapeutic Immunology & Infectious Disease, Department of Medicine, University of Cambridge, Cambridge, UK

**Keywords:** Antimicrobial use, Surveillance, Human medicine, Veterinary medicine, Vietnam, European Union

## Abstract

The accurate assessment of antimicrobial use (AMU) requires relating quantities of active ingredients (AAIs) with population denominators. These data can be used to prioritize potential sources of selective pressure for antimicrobial resistance and to establish reduction targets. Here, we estimated AMU in Vietnam (human population 93.4 M in 2015), and compared it with European Union (EU) data (population 511.5 M in 2014). We extrapolated AMU data on each key animal species and humans from different published sources to calculate overall AMU (in tonnes) in Vietnam. We then compared these data with published statistics on AMU in the European Union (EU). A total of 3838 t of antimicrobials were used in Vietnam, of which 2751 (71.7%) corresponded to animal use, and the remainder (1086 t; 28.3%) to human AMU. This equates to 261.7 mg and 247.3 mg per kg of human and animal biomass, compared with 122.0 mg and 151.5 mg in the EU. The greatest quantities of antimicrobials (in decreasing order) were used in pigs (41.7% of total use), humans (28.3%), aquaculture (21.9%) and chickens (4.8%). Combined AMU in other species accounted for < 1.5%. These results are approximate and highlight the need to conduct targeted surveys to improve country-level estimates of AMU.

## Main text

Antimicrobial resistance (AMR) in bacterial pathogens is now firmly recognized as major global health problem [1]. AMR arises as a direct consequence of antimicrobial usage (AMU) in humans and animals and resistant organisms and AMR-encoding genes are capable of crossing species barriers [2]. Therefore, the emergence and transfer of AMR means that control solutions need to be conducted from a ‘One Health’ perspective [3]. However, if we are to reduce AMR we need accurate estimates of where the majority of AMU occurs. Sustained surveillance and monitoring of AMU are widely acknowledged as critical components of the fight against AMR and one of the strategic priorities of the AMR Global Action Plan (GAP) [4].

There is considerable uncertainty regarding AMU in different animal species and humans in most countries. This knowledge gap is due to the absence of reliable AMU data in humans and animals and ill-defined animal population denominators. Many higher income countries, such as those within the European Union (EU), regularly publish their data on AMU in humans and animals, and relate these values to denominator populations in terms of biomass [5]. Conversely, the majority of low- and middle-income countries (LMICs) do not regularly collect and report equivalent AMU statistics.

Recently, the World Organization for Animal Health (OIE) estimated that worldwide, on average 168.7 mg of antimicrobial active ingredients (AAIs) were used to raise 1 kg of animal biomass [6]. Although the report does not include between-country- or species-specific data, it shows however considerable differences between different OIE regions. However, this report did not indicate which animal production sectors are responsible for the largest degree of AMU. Such data are essential for estimating where AMR is most likely to be generated and maintained and pivotal for policy makers to set reduction targets. Here, by integrating various data sources, we aimed to estimate AMU in humans and different animal populations in Vietnam. These data were compared against available human and animal AMU statistics from the EU.

Human biomass in Vietnam was calculated using 2015 population data stratified by age [7]. Adult (> 18 years-old) body weight was taken from published Figs. (58.4 kg males; 50.8 kg females) [8]. For non-adult age-gender strata, we assigned bodyweights to US populations [9], after adjusting for the difference in body mass between populations in the two countries. This was achieved by applying the correction factors of 0.642 and 0.651, which represent, respectively, the ratios of weights of adult males and adult females in the two countries. The total biomass of terrestrial animals in Vietnam was calculated from official statistics [10] following the approach used by the OIE [6] that combined data on the number of slaughtered animals and standing populations. For aquaculture (farmed fish and shellfish), production data broken down by type of market (domestic, export) (2016) were used [11].

Data on human AMU in Vietnam were extracted from a multi-country survey in hospitals and the community [12]. The reported number of Defined Daily Doses (DDD) (per 1000) were converted to weight of antimicrobial active ingredient (AAI) using the four most common administered antimicrobials (ceftriaxone, ampicillin, azithromycin and levofloxacin). The daily consumption data was extrapolated for a whole year (365 days).

For pigs, chickens, and aquaculture (all aquatic species combined) data on AMU were obtained from quantitative published surveys [13–16]. Data on on AMU through consumption of commercial feed (i.e. antimicrobial growth promoters) were extrapolated from a survey of 1462 pig and chicken commercial feeds in Vietnam [17]. Antimicrobial consumption in aquaculture was extrapolated from a previous study [18], assuming that, on average, antimicrobial products have a 20% strength (weight of AAI related to total weight of product) based on the same study. For ruminants (bovines, buffaloes, sheep, goats) data on AMU in Japan (a high-income country in Asia) for 2010 were used [19]. For non-chicken poultry species (ducks, Muscovy ducks, geese and quails) the authors could not find any published data. AMU was, therefore, conservatively estimated as 50% of that reported in chickens, based on the authors’ field experience. We excluded companion animals and equines since no AMU data are available. Best and worst-case AMU scenarios (i.e. lowest and highest AMU) were calculated for all species: for poultry species, upper and lower limits were calculated based on ±25% of the final AMU estimate. For ruminants, the lower limit of AMU was taken from Japanese cattle AMU statistics [19]. The upper limit was set at 50% higher than this estimate; for our summary estimations we used the intermediate value between these two limits. We compared the resulting AMU data with those published in the second ECDC/EFSA/EMA Joint Report on AMU (data for 2014), corresponding with AMU data in relation to the total biomass of terrestrial animal species in 28 EU countries [5] as well as with the Third World Organization for Animal Health (OIE) Report [6] (data for 2015).

**Fig. 1:**
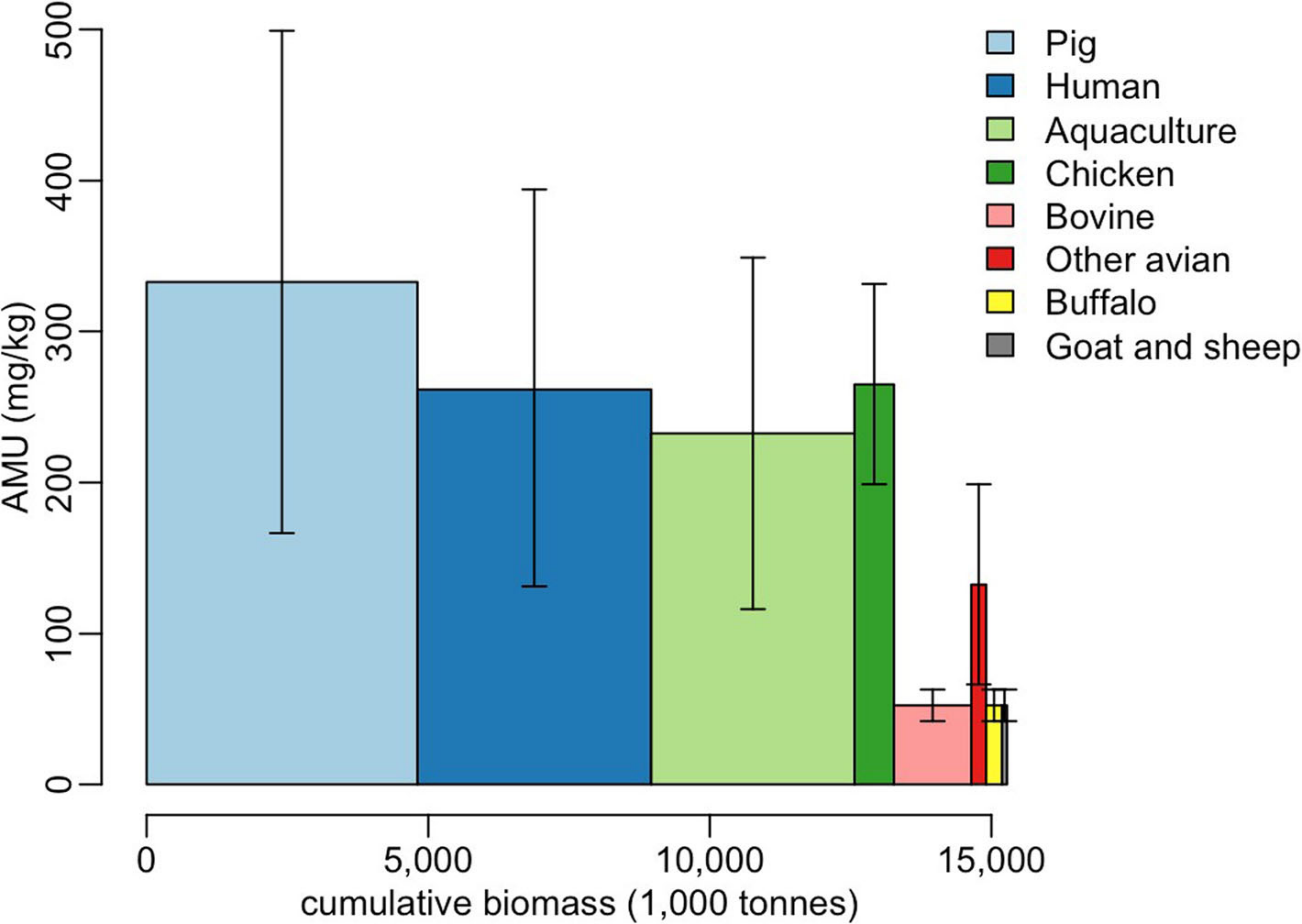
Two-dimensional diagram representing the estimated annual amounts (areas of bars) of antimicrobials used in each of species (including humans) in Vietnam, and whether these quantities are more affected by the total biomass (width of bars) or the intensity of AMU (height of bars). Bars are sorted from higher to lower overall AMU. AMU = Antimicrobial use. The vertical lines represent the range between best- and worst-case scenarios. ‘Other avian’ includes ducks, muscovies, geese and quails

**Fig. 2:**
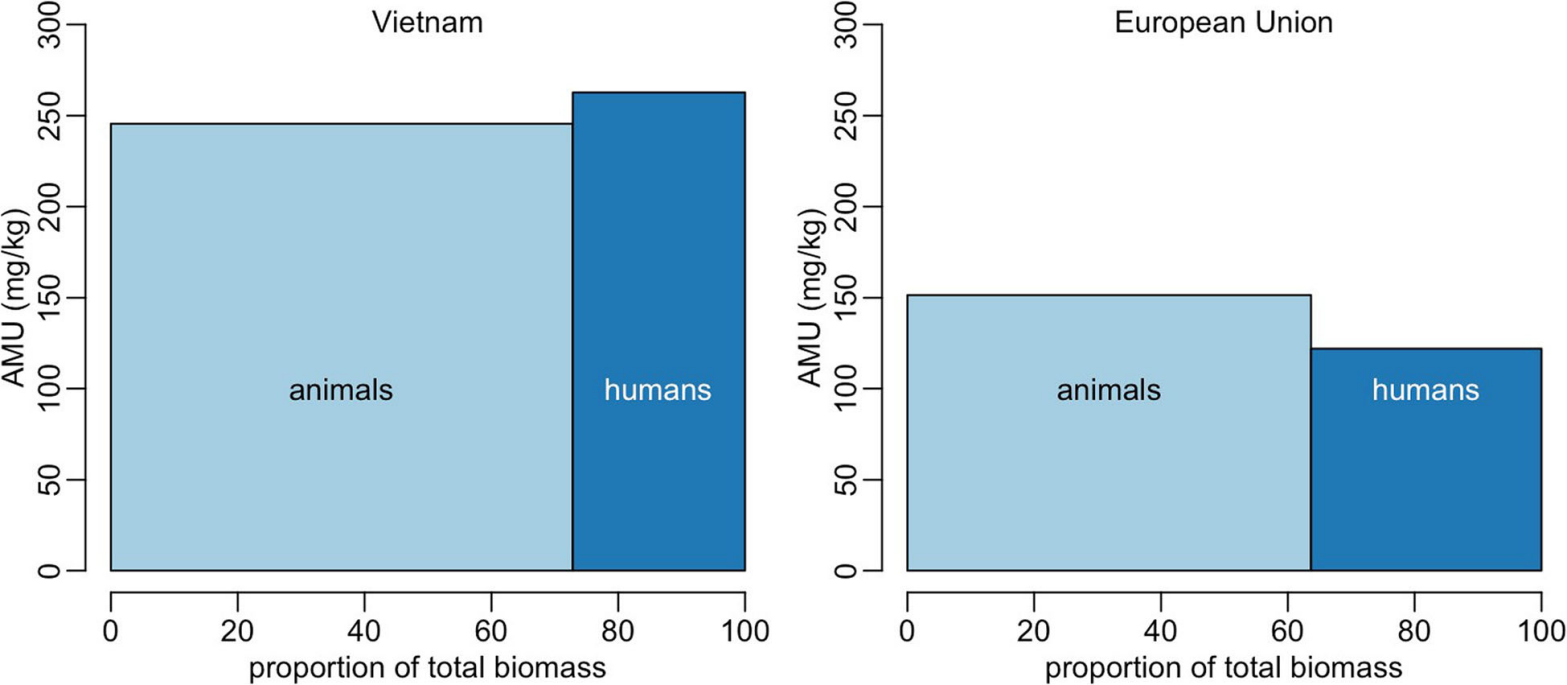
Two-dimensional diagram showing the relative annual amounts of AMU in the European Union (2014) and in Vietnam. In order to render comparison between European Union and Vietnam possible, the biomasses of animals and humans on the *x*-axis have been scaled to proportions. AMU = Antimicrobial use

Our estimates of human and animal biomass in Vietnam from the above calculations are 4153 and 11,125 thousand tonnes, respectively (Table 2 in Appendix 1 and Table 1). Estimates of AMU showed that in 2015, a total of 3842 t of antimicrobials were used in Vietnam, of which 2751 (71.7%) was associated with animal use, and the remainder (1086 t; 28.3%) corresponded to human AMU. The greatest quantities of antimicrobials (in decreasing order) were used in pigs (41.7% of total use), humans (28.3%), aquaculture (21.9%) and chickens (4.8%). Combined AMU in other species accounted for < 1.5% (Table 1 and Fig. 1). We estimate that, in total, 261.7 mg (131.4–394.3 mg) of AAI were administered per 1 kg of human and 247.3 mg (130.3–364.3 mg) per 1 kg of animal in Vietnam. The corresponding figures from the EU were 122.0 mg/kg and 151.1 mg/kg in humans and animals, respectively (Fig. 2).

**Table 1:** Calculation of total annual AMU in each animal production type

Category	Sub-category	No. of animals	Type of data^a^	Weight unit (kg)	Annual bodymass (kg)	AMU^b^ (mg per kg)	AGPs in commercial feed (mg per kg)	Total AMU (mg per kg)	Total AMU (tonnes)
Swine	Breeding pigs	4,128,032	Census	240	990,727,726	46.1^1^	286.6^2^	332.7	329.6
	Slaughter pigs (except breeders)	48,567,582	Production	78.6	3,817,411,914	46.1^1^	286.6^2^	332.7	1270.1
Poultry	Chickens	88,777,000	Production	1.8	699,798,600	187.7^3, 4^	77.4^2^	265.1	185.5
	Ducks	101,931,884	Production	2	203,863,767	93.9^5^	38.7^5^	132.6	27.0
	Muscovies	17,652,638	Production	3.2	56,488,440	93.9^5^	38.7^5^	132.6	7.5
	Geese	641,212	Production	3.2	2,051,877	93.9^5^	38.7^5^	132.6	0.3
	Quails	13,526,147	Production	0.13	1,758,399	93.9^5^	38.7^5^	132.6	0.2
Bovine	Breeding bovines	3,472,891	Census	325	1,128,330,008	52.4^6^	0.0	52.4	59.1
	Slaughter bovines (except breeding animals)	1,220,131	Production	200	244,026,240	52.4^6^	0.0	52.4	12.8
Buffalo	Breeding buffaloes	378,549	Population	500	189,274,500	52.4^6^	0.0	52.4	9.9
	Slaughter buffaloes	297,216	Production	300	89,164,711	52.4^6^	0.0	52.4	4.7
Sheep	Breeding animals (est.)	26,901	Census	75	2,017,556	52.4^6^	0.0	52.4	0.1
	Number slaughtered (except breeders)	64,368	Production	75	4,827,600	52.4^6^	0.0	52.4	0.3
Goats	Breeding animals (est.)	444,411	Census	75	33,330,833	52.4^6^	0.0	52.4	1.7
	Number slaughtered (except breeders)	699,515	Production	75	52,463,597	52.4^6^	0.0	52.4	2.7
Aquaculture	All species (domestic)	–	Production		835,000,000	477.1^7^	–	477.1^7^	398.5
	All species (export)	–	Production		2,775,000,000	159.1^8^	–	159.1^8^	441.4
All animals					11,125,535,768				2751.4

*AMU* Antimicrobial use, *AGPs* Antimicrobial growth promoters (in commercial feed)

^a^Data derived from official country statistics [10, 11]. ‘Census’ refers to ‘No. standing animals’, ‘Production’ refers to ‘No. of slaughtered animals’, except for aquaculture, where it refers to ‘No. of kg produced’

^b^Excluding antimicrobial growth promoters in commercial feed; ^1^ Nguyen et al. (2016) [15]; ^2^ Van Cuong et al. (2016) [17]; ^3,4^ Average of two studies: Carrique-Mas et al. (2014) [13] and Cuong et al. (2019) [14]; ^5^ Based on 50% of quantities used in chicken production; ^6^ Hosoi et al. (2014) [19]; ^7^ Pham et al. (2015) [16]; ^8^ Assuming that AMU for export production is 1/3 of the magnitude of AMU for domestic production

Here, using a combination of available statistics alongside published AMU survey and extrapolation data, we estimated AMU related to biomass in humans and animal production in Vietnam. Our results suggest that in this country pig production and aquaculture should be the main target if the country aims to reduce its AMU footprint in animal production. AMU in humans in Vietnam (32.0 DDD per 1000 inhabitants per day) ranks higher than in most countries in the EU. These human data were generated using limited retail surveys [12]. However, EU countries such as Romania, Greece, France, Spain, and Ireland featured a higher magnitude of AMU (in terms of DDD related to population) than Vietnam. A recent report from Thailand, a LMIC country which is more comparable to Vietnam, estimated that in 2017 a total of 53.0 DDD per 1000 inhabitants per day were used in 2017 [20]. The Thai study used surveillance data on declared quantities of antimicrobials, which is a compulsory requirement for companies trading with antimicrobials in that country.

Whilst these are the first specific calculations for AMU in Vietnam, there is a considerable uncertainty around these estimates due to the lack of reliable data. For example, AMU data in humans, pigs, and aquaculture originate from single studies, all conducted prior to 2015. Furthermore, there are no data whatsoever on AMU in non-chicken poultry species and ruminants. The situation is likely to be even worse in other LMICs where there are practically no AMU data in any production sector.

Since different animal types are raised over variable periods, the same magnitude of AMU related to body mass may have different implications for the development and maintenance of AMR For example, in Vietnam chickens are raised over a period ranging from 1 to 5 months, compared with 5–8 months for pigs. The implications of this need to be further investigated.

Because of its relative simplicity, we propose to regularly (i.e. annually) estimate/update quantities of antimicrobials used in relation to body mass as a first step to develop a fully-fledged AMU surveillance system. These estimates could be fine-tuned by conducting targeted surveys tailored to different production types (i.e. meat chickens, layers, breeders, fattening pigs, etc.). It may also be necessary to differentiate the extent of AMU by level of intensification of the production system (i.e. backyard, small-scale, large-scale, industrial), as different systems require variable quantities of antimicrobials. It has been shown that in the Mekong Delta of Vietnam smaller chicken farms tend to use more antimicrobials [13]. Lastly, it would be desirable to incorporate detailed information regarding the classes and formulations of antimicrobials used, since there is a great variability regarding the strength of different antimicrobial products and their impact on development of AMR.

In conclusion, in the absence of reliable statistics on sales of AAIs, the challenges of monitoring AMU in animal production in LMICs such as Vietnam can be overcome by the use of innovative approaches that maximize the use of existing animal population statistics and AMU data. These estimates should help elucidate secular changes in AMU and help refine policies and interventions aimed at reducing AMU at country level.

## Data Availability

The data presented and analysed here have all been extracted from publicly available data sets and publications cited in the body text.

## Appendix 1

Table 2

**Table 2:** Estimation of human bodymass of the Vietnamese population from population pyramid, adult bodyweight and age-gender specific bodyweights

Age (years)	Total population			Average weight (US) (kg)		Average weight (Vietnam)^a^ (kg)		Estimated bodymass (kg)		
	Males	Females	Total	Males	Females	Males	Females	Males	Females	Total
> 18	32,448,992	32,448,992	64,897,984			58.4	50.8	1,895,021,133	1,648,408,794	3,543,429,926
15 to 18	3,582,536	3,390,322	6,972,858	64.4	54.4	41.4	35.4	148,234,592	120,066,219	268,300,812
10 to 14	3,406,698	3,207,976	6,614,674	39.9	41.5	25.6	27.0	87,333,258	86,668,284	174,001,542
5 to 9	3,774,596	3,446,644	7,221,240	22.9	22.4	14.7	14.6	55,536,575	50,260,341	105,796,916
0 to 4	4,078,564	3,662,281	7,740,845	12.5	12.0	8.0	7.8	32,755,967	28,609,739	61,365,706
			93,447,601					2,218,881,525	1,934,013,377	4,152,894,902

^a^Estimated from US data after applying a correction factor 0.642 (males) and 0.651 (females)

## Appendix 2

Table 3.

**Table 3:** Estimation of weight of antimicrobial active ingredient from antimicrobials consumed by humans

Antimicrobial class	Antimicrobial	No. DDDs (per 1000 inhabitants per day)	Dose in g (for a typical inhabitant)	Daily amount of AAI (per 1000 inhabitants) (g)
Cephalosporin	Ceftriaxone	8	1.5	12
Broad-spectrum beta lactam	Ampicillin	8	1.5	12
Macrolide	Azithromycin	8	0.5	4
Oral fluoroquinolone	Levofloxacin	8	0.5	4
All				32
